# Variability of the Center of Mass in Trained Triathletes in Running After Cycling: A Preliminary Study Conducted in a Real-Life Setting

**DOI:** 10.3389/fspor.2022.852369

**Published:** 2022-06-06

**Authors:** Stuart A. Evans, Daniel James, David Rowlands, James B. Lee

**Affiliations:** ^1^SABEL Labs, Charles Darwin University, College of Health and Human Science, Darwin, NT, Australia; ^2^School of Engineering, Griffith University, Nathan, QLD, Australia

**Keywords:** accelerometer, center of mass, cycling, running, biomechanics, Sprint triathlon

## Abstract

While the sport of short-distance (Sprint) triathlon provides an opportunity to research the effect of the center of mass (CoM) when cycling and running, much remains to be done. The literature has failed to consistently or adequately report how changes to hand position influence subsequent running as inferred by the magnitude of CoM acceleration. The demands of cycle training in a drops and aerodynamic position followed by running remain unquantified in Sprint Distance triathlon. Thus, far data collected indicate that the cycle to run transition (T2) is important for overall race success. While many age-groupers participate in Sprint Distance triathlon, the lack of T2 based research make comparisons between cycle hand position and ensuing running difficult. The motion of the human body when cycling and running in triathlon can be described by the motion of its CoM in a local coordinate system. Unobtrusive wearable sensors have proven to be an informative resource to monitor the magnitude of CoM accelerations in running. However, the extent to which they are used in cycling is unclear. Therefore, the aim of the present study was to analyse the temporal magnitudes of CoM acceleration when cycling position and cadence is changed and to analyse these effects on running after cycling. Ten recreational triathletes completed two 20 km cycling trials at varied cadence in a drops position (parts of the handlebars that curve outward, Cycle_Drops)_ and an aerodynamic position (arms bent, forearms parallel to the ground, Cycle_Aero_) immediately followed by a 5 km run at self-selected pace. Torso kinematics by way of CoM acceleration magnitude were captured in a typical training setting using a triaxial accelerometer. CoM acceleration was quantified in m/s^2^ and variability was measured by the coefficient of variation (CV) and root mean square (RMS). Results from Cycle_Aero_ indicated that acceleration of the CoM in longitudinal (CV = 1%) and mediolateral directions (CV = 3%) was significantly reduced (*p* < 0.001) compared to Cycle_Drops_. As for rate of perceived exertion (RPE), a significant difference was observed with triathletes reporting higher values in Cycle_Aero_ alongside a greater CoM acceleration magnitude in the anteroposterior direction. The CoM varied significantly from Run_Aero_ with less longitudinal (CV = 0.2, *p* < 0.001) and mediolateral acceleration observed (CV = 7.5%, *p* < 0.001) compared to Run_Drops_. Although greater longitudinal acceleration was observed in the initial 1 km epoch of Run_Aero_, triathletes then seemingly adjusted their CoM trajectory to record lower magnitudes until completion of the 5 km run, completing the run quicker compared to Run_Drops_ (22.56 min^1^ ± 0.2, 23.34 min^1^ ± 0.5, *p* < 0.001, CV = 1.3%). Coaches may look to use triaxial accelerometers to monitor performance in both cycling and running after cycling.

## Introduction

Triathlon entails a sequential swim, cycle and run that is achieved over a variety of distances. The disciplines of cycling and running are the most time consuming regardless of distance (Cejuela et al., 2013). However, descriptions of incoordination are commonly reported among triathletes of all levels when moving from cycling to running (Heiden and Burnett, 2003). This leads to a potential competitive advantage to those that can minimize the presence of impaired movement coordination (Walsh, 2019). In this regard, further research concerning the effect of the multi-disciplinary cycle to run component is warranted.

Triathlon has been shown not to be “the sum of its component sports” since the neuromuscular alterations to cycling interfere with those elicited by running (Bonacci et al., 2009; Millet et al., 2009). Yet little research that can assist the triathlete to train in an optimal, Sprint Distance-specific, manner has been published. The training that is involved in preparation for the shorter Sprint Distance format (750 m swim, 20 km cycle, 5 km run) has been insufficiently quantified. Few detailed investigations of how changes in hand position whilst cycling may be reflected by changes in torso motion are available. This is relevant for coaches given that a lowered torso position when cycling can reduce frontal area and drag.

The torso (trunk) of the body includes the spine, hip and pelvis, whereas the torso muscles can be defined as those supporting the lumbopelvic-hip complex (Borghuis et al., 2008). The torso muscles provide proximal stability for distal mobility that involves more than muscles directly attached to the spine and pelvis (local and global stabilizers) (Panjabi, 1992). For instance, most of the prime movers of the upper- and lower extremities (e.g., the gluteal and hamstring muscles, latissimus dorsi) are attached to the torso. The torso and center of mass (CoM) transfer and control force and motion in an integrated kinetic chain and are crucial in both cycling and running. In this way, the goal of lower-limb movements is the forward translation of the body system, which itself can be mechanically represented by its CoM.

The CoM of a distribution of mass is the unique point in space whose linear acceleration is determined only by the total external force acting on the system, without effects due to internal forces (Goldstein et al., 2002; Adesida et al., 2019). When applied to the CoM, such external force causes a linear acceleration without resulting in whole-body angular acceleration. Because running after cycling is an unavoidable phenomenon, a question arises as to how cycling affects CoM acceleration in both cycling and running in triathletes. The trajectory of the CoM as the triathlete cycles in different positions, and the extent to which this is mirrored by changes to CoM acceleration in subsequent running, has not been explored in Sprint Distance triathlon.

Holding the handlebars that curve outward (the drop bars) helps reduce torso angle. A reduced torso angle is commonly associated with greater aerodynamics and a lowered center of gravity when cornering or descending. However, the strength of the relationship between torso stability and/or endurance when cycling in different hand positions is inconclusive (Saeterbakken et al., 2021). Thus, minimal examination of the extent to which cycling in either a drops position or an aerodynamic position has on CoM acceleration during both cycling and running has occurred. This is surprising given the importance of the torso relative to stabilization of the spine and maintenance of posture. Both torso and hand position are important considerations in triathlon as cycling efficiency has been observed to worsen as the torso angle drops (Fintelman et al., 2014).

Parameters such as movement speed, footwear, expertise, and fatigue impact CoM variability (Jordan and Newell, 2008; García-Pinillos et al., 2020). Yet the characterization of CoM variability relative to torso motion in Sprint Distance cycling and running remains unclear. To illustrate this concept, one can view two bicycle positions frequently used in Sprint Distance triathlon. The first, classified as the drops position, denotes holding on to the parts of the handlebars that curve outward, with the hands normally positioned directly behind the brake levers. The drops position can be described as cycling with palms placed on the drop bars near or parallel to the ground. The second position is known as the aerodynamic (aero position). The aerodynamic bar (aero bar) is an extension that is attached to a road bike that places the triathlete in a lowered position so that the thoracic spine is almost horizontal with the arms extended forward and elbows tucked in (Kyle, 1989; Ashe et al., 2003). In this regard, the torso is placed in greater flexion in the aero position when compared to the drops position. Crucially, the CoM is displaced, or accelerated, differently depending on the cycling hand position that is used by the triathlete.

The ability to proficiently link cycling and running segments has been quantified as integral to race performance (Millet and Vleck, 2000; Cuartero and Cejuela, 2021; Sousa et al., 2021). Nevertheless, transitioning from cycling to running in triathlon is complex and has been observed to cause biomechanical adaptations (Millet and Vleck, 2000; Chapman et al., 2008). Examples of such adaptations include differences in torso gradient (Hausswirth et al., 1997) and the vertical (longitudinal) acceleration of the CoM (Evans et al., 2020). Therefore, altering body position during cycling can result in significant changes to both lower extremity kinematics and to neuromuscular control patterns (Silder et al., 2011; Evans et al., 2021). In this sense, cycle and run performance can potentially be improved by modifying components of the bicycle-athlete interface.

Individual torso angle and the seat tube angle of the bicycle are both known to have a potentially beneficial effect on cycling and running kinematics (Ricard et al., 2006; Fintelman et al., 2014). Despite this, the research on these issues, as it relates to Sprint Distance triathlon, remains insufficient. For instance, efficient running requires postural stability that can enable coordinated movement of the thorax and pelvis, which then acts to minimize CoM motion (Preece et al., 2016). Greater acceleration can cause larger postural sway or reduced dynamic postural stability in the triathlete. This can result in less efficient running movement (Wiest et al., 2011). In triathlon, such dynamism and efficiency could be altered due to changes to the position of the hands during cycling. An alteration in running efficiency seems plausible since the triathlete tries to accelerate his/her body (represented by the CoM) forwards and upwards against gravity by pushing his/her body over the legs (Heise and Martin, 2001). As such, we believe that the influence of hand position in cycling and the corresponding impact on running in Sprint Distance triathletes represents an important variable that could improve performance.

The CoM trajectory can be described with respect to different reference points. For example, the origin of the lab coordinate system as a fixed reference point is one possible viewpoint (Möhler et al., 2021). Methods such as analyzing step-characteristics or ground-contact time lack the ability to quantitatively discriminate between subtle running differences (Weich et al., 2019). Body-worn sensors and wearable technology are attractive because of the potential to measure human movement unobtrusively, in the field, and at a comparatively low cost when compared to laboratory-based equipment (Lee et al., 2018). Nowadays, numerous and valid methods exist to observe and measure CoM motion, including the “sacral marker” method. However, the utility, reliability and effectiveness of sensor technology must be carefully considered when moving from a laboratory environment to one in the field.

With minimal information on the topic published thus far, the extent to which a Sprint Distance triathlete accelerates his or her CoM when cycling in a drops or aerodynamic position is unknown. Importantly, as cycling involves changes to cadence, how cadence alters the magnitude of CoM acceleration remains undetermined. The question arises as to whether cadence and cycle hand position alters CoM acceleration as well as how both impact running when considered from the mechanical vantage of the CoM and a local coordinate system. Therefore, the aim of the present study was to analyse the temporal activity of the magnitude of CoM acceleration when cycling hand position and cadence is changed and to analyse these effects on running after cycling.

## Materials and Methods

### Participants

Ten competitive recreational triathletes (7 male, 3 female) were recruited from a local triathlon club for this preliminary study (age: 32 ± 3.2 years; body mass: 71.4 ± 2.2 kg; weekly training frequency: 11.1 ± 2.3 hours; mean 5 km running pace obtained prior to testing: 4.25 per km (mm:ss) (3.5 m/s^1^) experience in Sprint Distance triathlon 8.1 ± 1.7 years). To be eligible, individuals were required to be ≥18 years of age. All participants gave written informed consent that followed the guidelines of the Human Research Ethics Committee board (HREC 030317) of Charles Darwin University. Participants were healthy and had no identified neuromuscular or musculoskeletal disorders at the time of the study. All participants were free from illness as established by the American College of Sports Medicine (2010) participant activity readiness questionnaire (PAR-Q). Participants were requested to refrain from vigorous exercise over the 24 h prior to testing and were instructed to preserve their standard diet.

### Research Protocol and Data Analyses

The triathletes were tested using their personal bicycles complete with both drop bars and clip-on aero bars in order to eliminate the effects of unfamiliarity. The purpose of this approach was to ensure the triathletes maintained the required cadences as specified in Table 1. The use of the triathlete's personal bicycles also limited the unfamiliarity of using different equipment.

**Table 1:** Cadence (in rev/min^1^) and running protocol performed for Experiment 1 and Experiment 2.

**Duration (Epoch)**	**Lap 1** **5 km**	**Lap 2** **10 km**	**Lap 3** **15 km**	**Lap 4** **20 km**	**Transition**	**Run 5 km**
Cadence	Self-Selected cadence	55–60 rev/min^1^	75–80 rev/min^1^	95–100 rev/min^1^	<60 s	Self-selected pace

The triathletes completed two experimental sessions, 1 week apart. The first experimental session began with measurements of cycle seat height, inseam leg length and seat tube angle (saddle height: 75 ± 0.3 cm; inseam: 73 ± 2 cm, and seat tube angle: 79° ± 0.1). A standard ergonomic and anthropometric tape measure (Seca 201 ergonomic circumference measuring tape, Hamburg, Germany) was used to measure seat height and inseam leg length by the same researcher. The inseam leg length was defined as the distance from the ischial tuberosity to the floor. The seat tube angle, being the angle of the seat tube relative to the horizontal plane, was manually measured by the principal author from behind the seat tube. Seat height was measured from the center of the pedal axle to the saddle top, with the pedal at the most distal end (Gregor et al., 1991).

**Figure 1:**
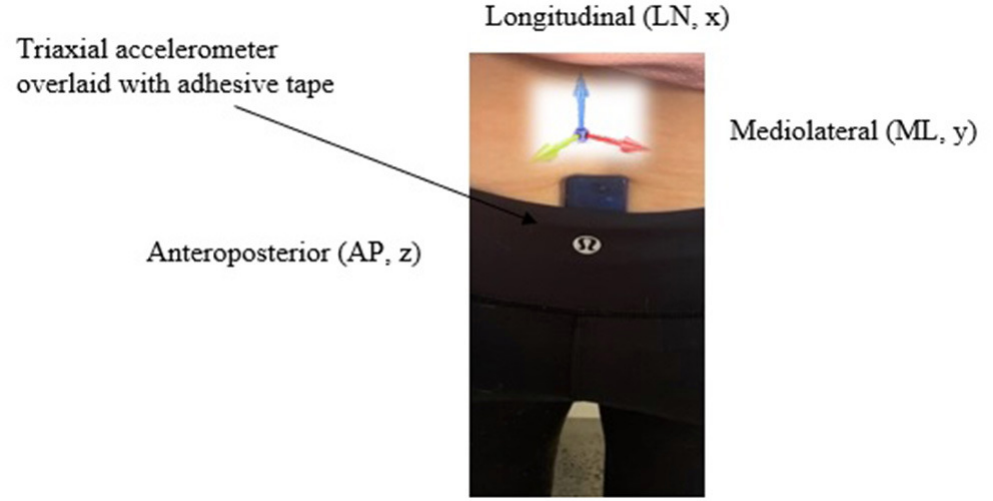
Depiction of orthogonal axes orientation and sensor used in study.

A single tri-axial accelerometer (52 mm × 30 mm × 12 mm, mass 23 g; resolution 16-bit, full-scale range 16 g: SABEL Labs, Darwin, Australia) was used to capture acceleration data in three orthogonal axes. The axes were defined as longitudinal (LN, x) mediolateral (ML, y) and anteroposterior (AP, z) and were aligned with anatomical axis and planes of motion as seen in Figure 1. Linearity and the subsequent categorization of the linear variables for each sensor was applied with gravitational (*g*) acceleration (9.8 m/s^2^) used as a reference source. The data were then manually scaled into m/s^2^. The accelerometer was located over the participant's spinous processes, defined as the lumbar vertebrae position 5 (L5) and sacrum vertebrae position 1 (S1) (James, 2006). The accelerometer was then securely fastened to participants using double sided elastic adhesive tape to diminish unwanted movement (Adesida et al., 2019). In this paper, the body system as a whole is represented, from a mechanical standpoint, by its CoM.

**Figure 2:**
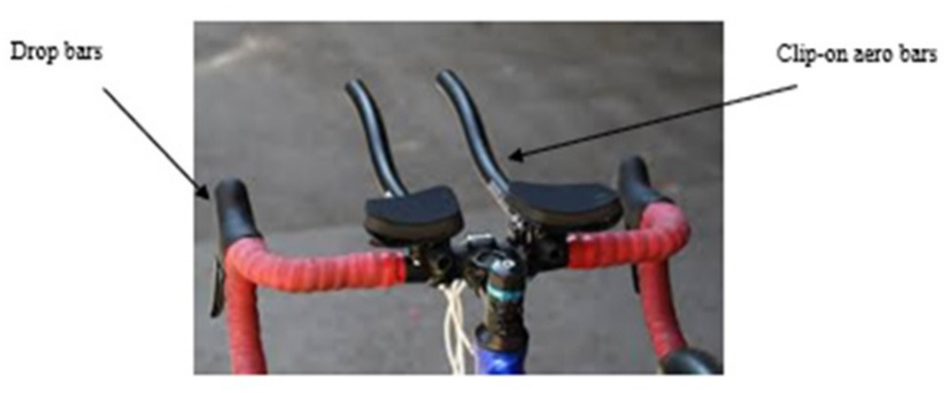
Representation of drops and aerodynamic positions.

The first experiment (session 1) commenced with a 10 min warm up on the bicycle that was performed at a self-selected cadence and body position prior to data collection and before the triathlete was randomly assigned to either a drops (Cycle_Drops_) or to an aero (Cycle_Aero_) cycling position (Figure 2). The triathletes were able to change gear by using the right and left-handed shifter controls on their respective cycles. In this regard, the right-hand shifter controlled rear wheel shifting whereas the left-hand gear shifter controlled the front. This process involved the triathlete pushing the inner, smaller gear paddle either left or right to drop (shift) to a smaller chainring or right to a larger chainring. The gear shifters were integrated into the cycle frame. The triathletes were unable to shift gears using the aerobar device. Zipp (Indianapolis, USA) Vuka Carbon Straight 22.0 mm aerodynamic bar extensions were used.

The triathletes then performed 4 × 5 km laps of cycling at a varied yet progressively increased cadence in either a Cycle_Drops_ or Cycle_Aero_ position. Once finished, a timed transition period of 60 s was permitted to allow the triathletes to change from cycling shoes with cleats into common athletic/running shoes as is common during Sprint Distance triathlon. Triathletes then commenced a 5 km run performed at self-selected pace (Run_Drops_, Run_Aero_). The second experiment (session 2) commenced with the triathletes performing the same 4 × 5 km laps of cycling at a varied yet progressively increased cadence on the same route, in either a Cycle_Drops_ or Cycle_Aero_ position (Table 1). This was followed by the same timed 60 second transition period and 5 km run performed at self-selected pace. The distances for cycling and running were chosen based on a customary Sprint Distance triathlon competition. The field-based location that was selected for both experiments was based on participant familiarity and it being a typical training setting.

To determine the effects of cycling on running, Chapman et al. (2007) designed a moderate-intensity protocol aimed at minimizing the impact of fatigue. In particular, they identified the typical ranges of cadence from data collected when elite triathletes competed at an international level. These data were then used to create a cycling protocol. This variable-cadence protocol devised by Chapman et al. (2007) has been used to identify the effect of changes to neuromuscular control and economy (Bonacci et al., 2011) and muscle recruitment patterns (Chapman et al., 2008) within running after cycling on subsequent running performance. We believed that the use of this protocol was deemed satisfactory due to the triathlete's familiarity with the cadences. Additionally, the triathletes in our study had frequently trained using cadence variation. Secondly, we believed that cadence was suitable for Sprint Distance triathletes owing to its ease of measurement in that all the triathletes had fitted speedometers. Cadence was viewable via individual display meters mounted onto the triathletes' bicycles in order that each triathlete could monitor the appropriate revolutions per minute. The speedometers were manually calibrated by way of roll-out distance, defined as the distance the bike travels in a straight line through one full revolution of the pedal cranks in the biggest gear. Consequently, to accomplish the purpose of this preliminary study and to limit the influence of fatigue, the moderate-intensity protocol of Chapman et al. (2007) was used in our study.

To monitor the onset of fatigue, the Borg ratings of perceived exertion scale (RPE) 6–20 scale (Borg, 1998), where 6 means “no exertion at all” and 20 means “maximal exertion,” was used. The RPE scale was used by the triathletes for self-monitoring intensity to ensure that effort was kept within the moderate-intensity/somewhat hard range. The triathletes were requested to keep within an upper bound of 13–14 (defined as “somewhat hard”). The triathletes had previous experience of using perceptual RPE scaling. The triathletes verbally provided a number that corresponded to the Borg 6-20 scale at the conclusion of each 5 km cycle lap and at each 1 km of running. Northwave tri-sonic cycling shoes (Northwave, Via Levada, Pederobba TV, Italy) with fitted Shimano SPD-SL clipless pedals and yellow cleats with ~6° flotation and tightness were worn by all triathletes. Cleat position was aligned to the head of the first metatarsal, positioned directly above the pedal spindle with the foot placed laterally in the middle of the pedal (fore-aft). Participants wore a typical one-piece triathlon racing suit in both cycling and running experiments. Time was recorded in minutes and seconds (mm:ss) using a Sportline 240 Econosport manual stopwatch (Yonkers, New York City).

**Figure 3:**
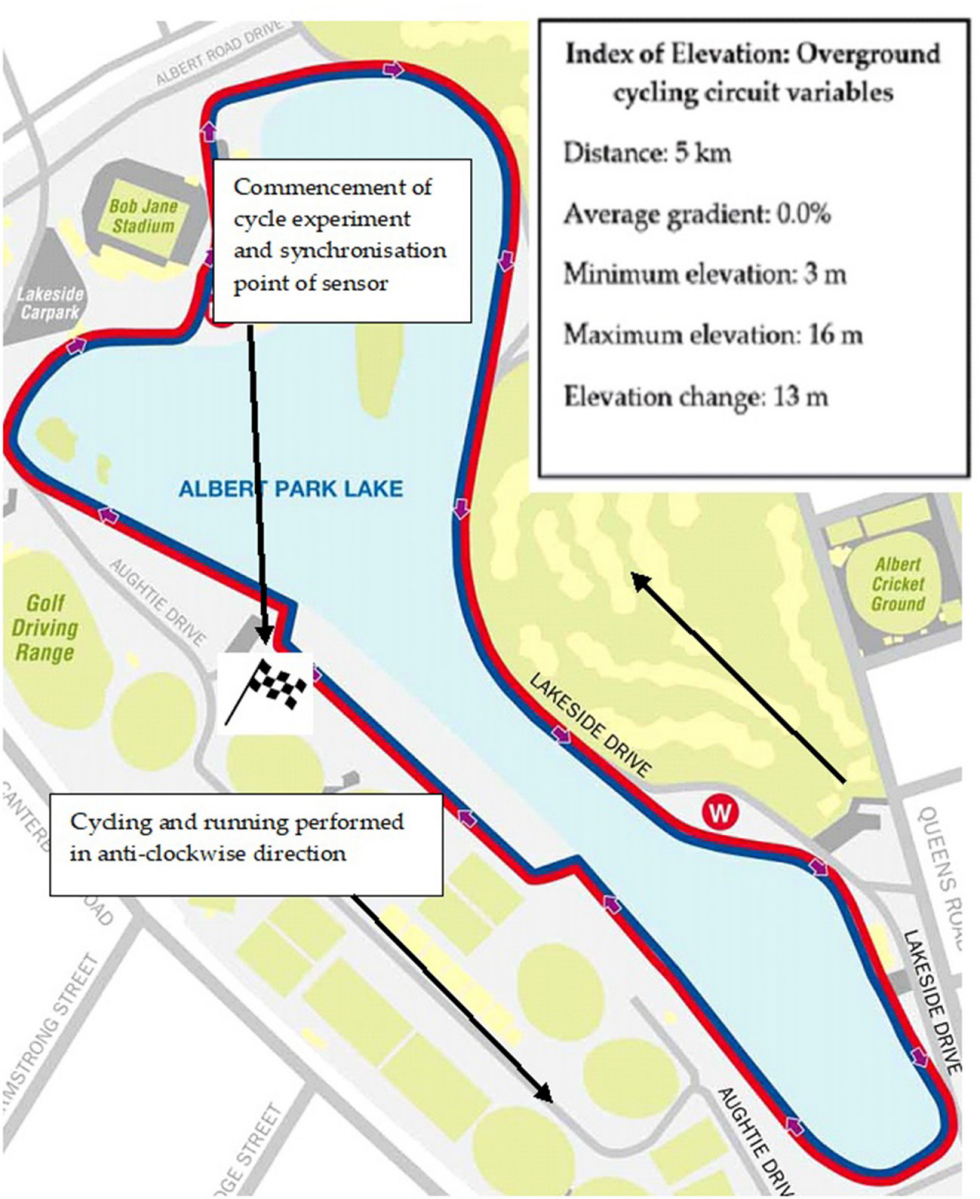
Map view and location of both experiments. The index of elevation contains the course variables experienced by the participants during both experiments. The average gradient across the 5 km circuit was 0%. Image retrieved from https://albertpark.com/albert-park/albert-park-lake/.

The 20 km randomized cycle protocol for experiment 1 and experiment 2 was accomplished on a predominately flat course (average gradient 0%) that is frequently used by both elite and recreational triathletes. The course was intentionally nominated due to its low level of technical difficulty. In this regard the course allowed for a representative evaluation of accelerometer data that was based on the triathletes' familiar training location. Triathletes were evaluated at the same time of the day (between 0700 and 0900), under similar environmental conditions (16–17°C, 60–65% relative humidity and a mean wind speed of 1.08 knots (kts) (0.56 m/s^1^) in a North-Easterly direction). A marked black and white checkered grid was etched on the asphalt course in order to signify when triathletes were required to change cadence. The checkered grid represented the start and end of one 5 km cycling lap. The cadence changes that were to be implemented by the triathletes were verbally communicated by the principal author when the front wheel of participants' bicycles contacted the checkered grid (Figure 3).

The triaxial accelerometer was controlled wirelessly by the principal author via a standard Hewlett Packard PC using a comprehensive MATLAB Toolkit (ADAT Toolbox, SABEL Labs, Darwin Australia). Sampling rates for both experiments of data capture were set as 100 Hz. Data were later downloaded from the accelerometer using a SABEL Sense software program (SABEL Sense 1.2 x64, SABEL Labs) via a CSV file. For each participant and on every occasion prior to commencing cycling, a static calibration of each device was performed (Lai et al., 2004). No filtering was applied to the data. For repeatability of measurement, the same sensor was used in both experiments. The signal vector of the x (LN), y (ML) and z (AP) CoM acceleration magnitude of each triathlete was calculated. The signal vector calculation was based on the mean triaxial acceleration recorded over a 60 second epoch of cycling and running, excluding the warm up, to minimize variability and obtain a true reflection of steady state cycling and running. The raw accelerometry signals were converted from CSV format and saved and exported to Microsoft Excel (Microsoft Corporation Redmond, Washington DC, USA version 4.90.4, build 6470.27615).

### Accelerometer Initialization and Setup

In this paper, torso accelerations of each local component were collected for each triathlete in order to examine the magnitude and trajectory of CoM acceleration in both cycling positions (i.e., experiment 1 and experiment 2) and the consequential influence on running. In this regard, the accelerations of the trunk and subsequent data output provided indicative measurements of the relative and local stability of the triathlete during both cycling and running. A lesser amount of acceleration magnitude equates to less unwanted movement, or greater trunk stability. Whilst the longitudinal and mediolateral axes were in different orientations for the cycling and running components, the accelerometer remained equivalent relative to each participant and local component. In this instance, the longitudinal axis pointed forward during cycling and pointed vertically during the run. The differences relative to each triathlete and change in body position was then analyzed in the accelerometer raw data. The local coordinates were therefore valid given that they considered the individual anthropometric variability of each triathlete. Therefore, it was decided that a local reference frame would still provide relevant information about the relationship between variables. It would be likely that a global reference frame would provide a similar relationship at the expense of extra computation and analysis. However, for the purpose of this preliminary study it was decided the local axis relative to the triathlete would show whether there was an effect of changing hand position when cycling.

**Figure 4:**
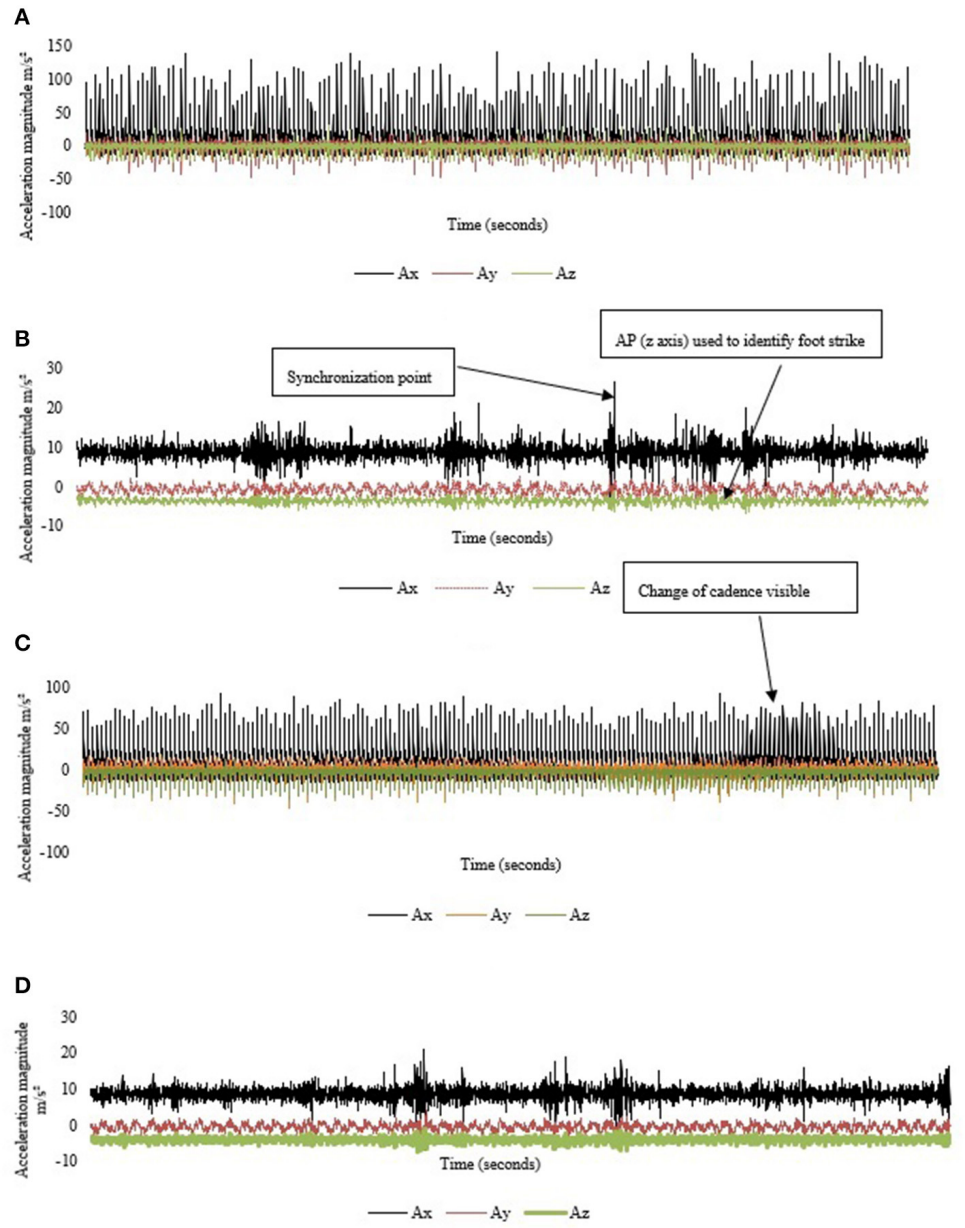
Example of raw signal vector magnitude of CoM acceleration during 60 s for one participant in **(A)** Cycle_Drop_; **(B)** Run_Drop_; **(C)** Cycle_Aero_; and **(D)** Run_Aero_. Where Ax is CoM longitudinal, Ay is CoM mediolateral and Az is CoM anteroposterior.

Self-selected pace when running was designated in that standardizing pace likely elicits changes in kinematic outputs. Specifically, people subconsciously choose self-selected pace due to that pace being the most mechanically efficient in regards to energy expenditure (Austin et al., 2018) and indications that running mechanics may alter above and below this pace (Lee et al., 2010a). Timing of temporal gait events of foot-strike and toe off were identified in the anteroposterior axis using a previously reported methodology (Lee et al., 2010b). At the conclusion of each 5 km cycling lap the accelerometer was manually synchronized by the principal author. This was to identify synchronization points in the raw data through *post-hoc* analysis. Consequently, in the current dataset cadence changes were observable due to the corresponding synchronization points. The coefficient of variation (CV) was calculated as a measure for each projection in the individual CoM acceleration directions (i.e., x, y and z) in cycling. Thus, the direction of acceleration magnitude could be quantified. The same process was repeated for the 5 km run (Figure 4).

### Statistical Analysis

Results are presented as mean values ± standard deviation. Descriptive statistics were calculated for both Cycle_Drops_ and Run_Drops_ and Cycle_Aero_ and Run_Aero_ for all the participants. A Shapiro–Wilk test was used to determine if the data were normally distributed. Despite the small sample size and preliminary status of this study, a parametric statistical procedure was selected. Statistical analysis was conducted using the Analyse-it statistical package (Leeds, United Kingdom, version 4.92). Center of mass acceleration data and variability between cycle hand position and running were quantified by the coefficient of variation (CV). Once completed, paired Student's *t-*tests were performed to identify potential differences between cycling hand positions and running conditions. To further understand the trajectory of CoM acceleration in each individual direction (i.e., x, y, and z) for both Cycle_Drops_ and Cycle_Aero_ relative to each cadence condition, a repeated measures analysis of variance (ANOVA) was performed. The RPE was implemented as a covariate. To estimate the continuous function of the magnitude of acceleration, the root mean square (RMS) value was determined. Within the running approach, the same method was used to analyse individual directions (i.e., anteroposterior, mediolateral and longitudinal direction) for both Run_Drops_ and Run_Aero_. The p value of *p* < 0.05 was considered statistically significant.

## Results

### Comparison of CoM Acceleration in Cycling Between Cycle_Drops_ and Cycle_Aero_

The results from Cycle_Aero_ indicated that longitudinal and mediolateral CoM acceleration magnitude was significantly reduced (*p* < 0.001) compared to Cycle_Drops_. As for RPE, a significant difference was observed with triathletes reporting higher values in Cycle_Aero_ compared to Cycle_Drops_ (Table 2).

**Table 2:** Magnitude of mean ± SD time series triaxial CoM acceleration in 20 km cycling in aero and drops positions (in m/s^2^).

**Direction**	**Aero cycling (mean + SD)**	**Drops cycling (mean + SD)**	***p***	**CV**
LN (m/s^2^)	2.84 ± 0.1	3.2 ± 0.1	<0.001*	1.0%
ML (m/s^2^)	−0.07 ± 0.1	−0.21 ± 0.1	<0.001*	3.0%
AP (m/s^2^)	−0.09 ± 0.6	−0.07 + 0.4	<0.001*	1.6%
RPE	38.10 ± 1.1	36.15 ± 1.1	<0.001*	>1%

*Where LN, longitudinal (x), ML, mediolateral (y); AP, anteroposterior (z) torso acceleration. RPE, ratings of perceived exertion*.

*p < 0.05.

During Cycle_Aero_, total CoM acceleration was significantly lower (*p* < 0.001) across 20 km cycling despite the highest RMS observed at both self-selected cadence and 55–60 rev/min^1^. Of note is that as cadence increased to 75–80 rev/min^1^ and 95–100 rev/min^1^, RMS decreased in both Cycle_Aero_ and Cycle_Drops_ compared to earlier cadence ranges (Table 3).

**Table 3:** Descriptive statistics for the magnitude of mean triaxial torso acceleration in cycling in aero and drops positions across all cadences (in m/s^2^).

**Variable**	**Lap 1**	**Lap 1**	**Lap 2**	**Lap 2**	**Lap 3**	**Lap 3**	**Lap 4**	**Lap 4**	**Standardized difference between means**
	**Aero**	**Drops**	**Aero**	**Drops**	**Aero**	**Drops**	**Aero**	**Drops**	
	**SSC**	**SSC**	**55–60 rev/min^**1**^**	**55–60 rev/min^**1**^**	**75–80 rev/min^**1**^**	**75–80 rev/min^**1**^**	**95–100 rev/min^**1**^**	**95–100 rev/min^**1**^**	
LN (m/s^2^)	2.79	3.04*	2.85	3.10*	2.77	3.06*	2.99	3.32*	0.09*
	± 0.1	± 0.3	± 0.2	± 0.1	± 0.4	± 0.5	± 0.6	± 0.4	± 0.9
ML (m/s^2^)	−0.6	−0.2*	−0.17	−0.23	−0.06	−0.20*	−0.02	−0.23*	−0.2*
	± 0.2	± 0.3	± 0.5	± 0.2	± 0.4	± 0.3	± 0.7	± 0.8	± 1.1
AP (m/s^2^)	−3.04	−2.14*	−2.98	−2.07*	−2.40	−2.11	−2.08	−1.61*	1.80*
	± 0.1	± 0.2	± 0.4	± 0.2	± 0.4	± 0.5	± 0.7	± 0.6	± 0.5
RMS	4.12	3.70	4.12	3.72	3.67	3.72	3.64	3.70	
RPE	8.1	7.5	9.6	9.3	9.8	9.5	10.6	10.2	0.4*
	± 0.1	± 0.1	± 0.2	± 0.1	± 0.1	± 0.2	± 0.1	± 0.3	± 0.1
Lap Time (mm:ss)	10.22	10.31	10.48	10.56	10.01	10.11	9.51	9.59	0.08*
	± 0.1	± 0.1	± 0.2	± 0.2	± 0.4	± 0.1	± 0.4	± 0.3	± 0.1

*Where LN, longitudinal (x), ML, mediolateral (y); AP, anteroposterior (z) torso acceleration*.

^*^SSC, self–selected cadence; RPE, ratings of perceived exertion; CV, coefficient of variation; RMS, Root Mean Square. Lap time given in minutes and seconds (mm:ss).

^*^p < 0.05 between aero and drops cycling.

### Comparison of the Magnitude of CoM Acceleration in Run_Aero_ and Run_Drops_

Results from Run_Aero_ showed that longitudinal and mediolateral CoM acceleration magnitude was significantly reduced (*p* < 0.001) compared to Run_Drops_. However, this was not the case when the magnitude of anteroposterior acceleration was considered, albeit with a non-significant result. Overall run completion times were quicker, and RPE was lower, for the Run_Aero_ condition compared to the Run_Drops_ condition (Table 4).

**Table 4:** Descriptive statistics for the magnitude of mean ± SD longitudinal and mediolateral timeseries torso acceleration in running after aero and drops cycling (in m/s^2^).

**Direction**	**Run Aero**	**Run Drops**	***P***	**CV**
LN (m/s^2^)	8.60 ± 0.1	8.81 ± 0.5	<0.001*	0.2 %
ML (m/s^2^)	−0.07 ± 0.1	−0.48 ± 0.1	<0.001*	7.5 %
AP (m/s^2^)	−3.10 ± 0.2	−2.91 ± 0.2	0.53	1.0 %
RPE	10.6 ± 0.2	11.5 ± 0.2	<0.001*	1.6 %
Mean run time (mm:ss)	22.56 ± 0.2	23.34 ± 0.5	<0.001*	1.3%

*Where LN, longitudinal (x); ML, mediolateral (y); AP, anteroposterior (z). RPE, ratings of perceived exertion. Lap time given in minutes and seconds (mm:ss)*.

^*^p < 0.05.

**Figure 5:**
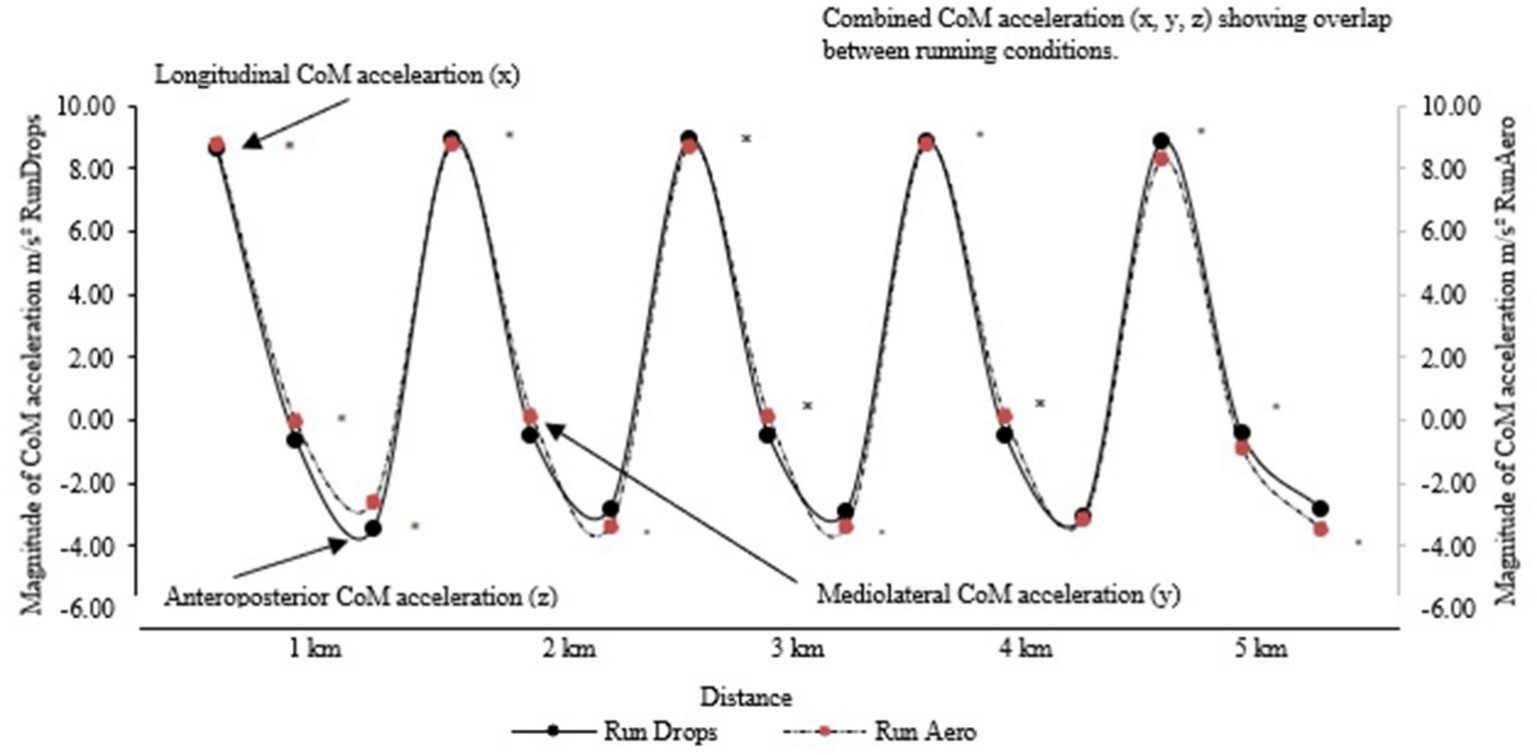
Mean sinusoidal time series representation of triaxial acceleration magnitude where LN = longitudinal (x), ML (y) = mediolateral (y); and AP (z) = anteroposterior torso acceleration in running after aero (dashed line) and drops (solid line) cycling for 10 participants (in m/s^2^). The resulting trajectories from each gait cycle were subsequently overlayed to observe possible differences between steps. The overlay plot is a time-based in which the mean magnitude was plotted as a function of time Axy(t) vs. (t). The mean triaxial acceleration magnitude was plotted for each 1 km for the designated duration. *p* < 0.05.

To visually distinguish between total CoM acceleration (x, y, z) in 5 km running after both Run_Drops_ and Run_Aero_, a timeseries representation of sinusoidal curves for all triathletes was created with a repeated measures ANOVA revealing no significant effect to total triaxial acceleration magnitude (*f* = 0.01, *p* < 0.05) (Figure 5).

From here, mean CoM acceleration was analyzed per 1 km of running to compare Run_Drops_ and Run_Aero_. Concerning Run_Aero_, it was found that the triathletes significantly reduced their longitudinal and mediolateral CoM acceleration compared to Run_Drops_. A significantly higher RPE was reported by the triathletes during Run_Drops._ Although greater longitudinal acceleration was detected in the initial 1 km of running after Run_Aero_, temporal magnitudes of longitudinal acceleration then abated with significantly faster km run completion times observed compared to Run_Drops_ (*p* < 0.001). No statistical difference were observed between the two running states in the anteroposterior direction (Table 5).

**Table 5:** Descriptive statistics for magnitude of mean ± SD longitudinal and mediolateral torso acceleration in running after aero and drops cycling (in m/s^2^).

**Direction**	**1 km run Aero**	**1 km run Drops**	**2 km run Aero**	**2 km run Drops**	**3 km run Aero**	**3 km run Drops**	**4 km run Aero**	**4 km run Drops**	**5 km run Aero**	**5 km run Drops**	**Standardized mean difference**
LN (m/s^2^)	8.82	8.66*	8.79	8.96	8.74	8.95*	8.79	8.89*	8.31	8.90*	0.17
	± 0.1	± 0.5	± 0.1	± 0.4	± 0.6	± 0.2	± 0.4	± 0.6	± 0.3	± 0.6	± 0.26
ML (m/s^2^)	−0.01	−0.60*	−0.16	−0.44*	−0.14	−0.50*	0.15	−0.46*	−0.83	−0.43*	0.4
	± 0.3	± 0.5	± 0.2	± 0.5	± 0.6	± 0.7	± 0.2	± 0.8	± 0.8	± 0.4	± 0.45
AP (m/s^2^)	−0.01	−0.50	0.29	−0.33	0.28	−0.40	0.28	−0.39	−1.47	−0.32	0.18
	± 0.6	± 0.4	± 0.5	± 0.4	± 0.5	± 0.3	± 0.5	± 0.2	± 0.2	± 0.4	± 0.61
RMS	8.82	8.69	8.80	8.98	8.75	8.97	8.80	8.91	8.48	8.92	

*Where LN, longitudinal (x); ML (y), mediolateral (y); AP, anteroposterior (z). RMS, root mean square*.

*p < 0.05.

## Discussion

Understanding the motion of the human body CoM when cycling and running in Sprint Distance triathlon is important in the efforts of providing triathletes with information to assist in performance. The aim of the present study was therefore to analyse the temporal activity of the magnitude of CoM acceleration when cycling hand position and cadence was changed, and to analyse the temporal CoM motion on running after cycling. Two commonly used cycling positions, namely the drops and aerodynamic position, were assessed. Both positions require a change in hand placement owing to the proximity of the drops and aerodynamic bars necessitating a change in torso position. This change in position resulted in a subsequent alteration to the magnitude of CoM acceleration. The same cadence protocol was used for cycling in a drops position and an aerodynamic position. Therefore, the impact of cadence on the magnitude of CoM acceleration was evaluated. In the following sections, we will discuss the findings of the Cycle_Drops_ and Cycle_Aero_ experiments and then provide comments on the methodological considerations concerning Run_Drops_ and Run_Aero._

### Body Position and Cadence Effects on CoM Acceleration Between Cycle_Drops_ and Cycle_Aero_

We analyzed the effects of the CoM trajectory by way of acceleration magnitude and the relative variability using the two aforementioned body positions when cycling; where the position of the CoM was described comparative to a local coordinate system. Firstly, our results add to the existing body of evidence showing that the arms play an active role in raising and lowering the torso in cycling (e.g., Stone and Hull, 1993). However, to the authors' best knowledge, no prior studies have investigated the CoM trajectory and acceleration magnitudes during the combined cycle to run discipline, notably with changes to hand position when cycling.

Concerning Cycle_Drops_, an increased magnitude of CoM acceleration for both longitudinal and mediolateral acceleration was observed despite the triathletes during this manifesting a lower RPE score compared to Cycle_Aero_. The greater mediolateral acceleration is likely caused by insufficient torso strength which itself could have resulted in more mediolateral movement whilst pedaling. Evidence to support this can be seen in a study by Rannama et al. (2017) in that a reduced level of core muscle strength can result in greater upper body movement. Thus, as cadence varied the demands on the triathletes' torso stability may have subsequently changed. Whilst the adoption of higher cadence resulted in triathletes completing each 5 km cycle lap quicker, further increases to mediolateral acceleration could have a greater detrimental influence on performance. Under such circumstances, a more conservative or balanced pacing strategy that allows reduced mediolateral movement may be beneficial for overall performance. However, this proposition is quite generalizable; therefore, its application to lower-limb performance requires additional analysis. Despite this, the existing cycle-specific literature suggests that a portion of lower-limb power acts to raise the CoM rather than to move the crank (Baker et al., 2002). Our results suggest that as cadence increased so did the magnitude of acceleration that was generated by the triathletes. The extent to which this occurred may relate to each triathlete's ability to maintain trunk stability when moving from high to low cadences. However, this assumption has not been verified.

As for the Cycle_Aero_ condition, the magnitude of anteroposterior acceleration was greater across all cadences when compared to Cycle_Drops_. The reasons for the differences in anteroposterior acceleration between the two cycle positions are not fully understood. Factors that affect the magnitude of anteroposterior acceleration may relate to the triathlete's total gross motion during Cycle_Aero_. Greater magnitudes of anteroposterior acceleration may have occurred if the triathlete applied greater force to the crank. The anteroposterior motion of the CoM could be construed as representative of the triathlete's overall gross motion when cycling in an aerodynamic position. Thus, when attempting to apply greater force to the crank, the effort may not be transferred effectively to performance output but instead to unwanted body movement. We suspect that in this scenario, movement of the CoM alters due to the need to maintain both cadence and torso control. The torso and its central position in the body, transfer and control force and motion in an integrated kinetic chain and is crucial in every athletic function (Kibler et al., 2006; Behm et al., 2010). Unfortunately, reports of the synchronous recording of motions of body segments and the CoM are rare (Saeterbakken et al., 2021), particularly when applied to cycling and Sprint Distance triathlon. More research is needed to better understand the complexity of cycle biomechanics that contribute to the development of CoM acceleration.

Throughout Cycle_Aero_, where the torso may have been positioned more horizontally relative to the top tube of the bicycle, the quadriceps may be placed at a disadvantage because of excessive shortening. The mechanical advantage/disadvantage is related to how far forward the triathlete is positioned. Jeukendrup and Martin (2001) state that an athlete being in the aero position may lead to an improvement in aerodynamic drag. However, consideration must be given to the triathlete's relative joint angle and muscular output. Whilst triathletes do not “freeze” in a single position when cycling, a field-based study is more representative of a training and competitive situation than is that of a laboratory environment. Considering that both Cycle_Aero_ and Cycle_Drops_ were associated with a typical training situation, it would be beneficial to monitor muscular output to examine possible relationships with CoM magnitude. This may represent an opportunity for additional analysis in order for coaches to assess the relationship between temporal accelerations of the trunk and muscular output in Cycle_Aero_ and Cycle_Drops._

At first sight, the changes in the magnitude of CoM acceleration are difficult to place into context of the literature given the lack of past CoM related studies and therefore data that is relevant to Sprint Distance triathlon. Nevertheless, it must be noted that cycling in the field often involves a change of torso position. In contrast, when a participant is asked to cycle in a straight line using an ergometer or similar device in a laboratory environment, the torso is kept over the wheels (or base of support). This torso position is arguably more attainable in the drops bicycle position given the wider hand position. Steering input can be provided by the triathlete directly via handlebars (steering torque) or through the self-stability of the bicycle due to the coupling of bicycle steer and roll (e.g., a bicycle leant to its side (roll) will cause a change in its steer angle) (Cain et al., 2016). This coupling alters the dynamic motion of the torso as body movements such as leaning left and right have a smaller effect than steering. Yet this dynamic coupling motion of the torso permits balance corrections by shifting the torso mediolaterally relative to the bicycle and base of support. The greater horizontal position of the torso in Cycle_Aero_ could have entailed smaller magnitudes of CoM acceleration whilst simultaneously causing increased gross movement. Future studies should be conducted on differences between straight line cycling and dynamic coupling in order to analyze the effects of this on the magnitude of CoM acceleration and the gross motion of the triathlete.

### Body Position Effects on CoM Trajectory After Cycle_Drops_ and Cycle_Aero_ and the Effects on Running

Our results corroborated those obtained by prior studies (e.g., Garside and Doran, 2000) in so far as we found that prior cycling alters running kinematics to varying aspects. Specifically, the common observation of a more flexed torso that arises in the initial stages of triathlon running compared to the control run only. The major effect we observed was a significant increase in mediolateral CoM acceleration in Run_Drops_ (Table 4). In contrast, despite a significant yet relatively minor amount of variability in the longitudinal axis, this was not the case during the initial 1 km of running. When running after Run_Aero_, a significant increase in longitudinal acceleration was initially observed compared to the same distance from Run_Drops_.

It appears that the triathletes were choosing different strategies during the initial 1 km of running based on if Cycle_Drops_ or Cycle_Aero_ was performed. In running, the goal of lower-limb movement is the forward translation of the body system, mechanically represented by its CoM (Tesio and Rota, 2019). In this sense the magnitude of longitudinal acceleration reduced from the 2 km Run_Aero_. These discrepancies might be due to the concomitant increases in mediolateral motion as the triathletes seemingly adjusted their running gait. On the other hand, as anteroposterior acceleration remained relatively stable during Run_Drops_ compared to Run_Aero_ it remains feasible that too much or too little acceleration in the sagittal plane can cause performance detriments. The magnitude of anteroposterior acceleration has been associated with braking motion upon foot strike (or ground contact) as the anteroposterior force component shows a typical slowing and propulsive phase. In contrast to the anteroposterior force component, the mediolateral force component is characterized by more variability (Munro et al., 1987). In this instance, one can suggest that a reduction to anteroposterior CoM acceleration magnitude could be beneficial. Otherwise, the mechanical inefficiency of having to overcome braking action affects performance. So far, our results suggest that a trade-off between longitudinal and anteroposterior CoM magnitude could be required when running from different cycling positions. Accordingly, this is not a one-time process as triathletes can attain different torso positions by undertaking adaptive training on their pedaling style and body flexibility, and this in turn will influence the CoM trajectory. However, most of the work to move the body system, its segments and its displacements, can be observed in the sagittal plane (Tesio et al., 1998). Running and cycling movements require the triathlete to remain predominantly in the sagittal plane. In this regard, a triathlete's total work to move his/her body would not be expected to change remarkably when the other anatomical planes are considered.

The increased CoM longitudinal acceleration could partially explain the increased 5 km running time observed in Run_Drops_ since triathletes would have to overcome increases in vertical loading rates and vertical ground reaction forces. In this study, a significantly different mean run time of 04.42 (mm:ss) per km (3.55 m/s^1^) was noted in Run_Drops_ compared to the quicker 04.35 (mm:ss) per km (3.64 m/s^1^) during Run_Aero_. Wille et al. (2014) found that the magnitude of CoM motion is associated with vertical ground reaction forces and braking impulse. However, an exaggerated decrease in vertical oscillation of the CoM (Gordon et al., 2009) has been linked to an increase in the metabolic cost of running. Considering the need to define the differences between oscillation and acceleration, the acceleration of the CoM can be considered as proportional to its displacement from its equilibrium position. For periodic motion such as running, as frequency is the number of oscillations per unit time, the CoM begins to accelerate in the positive × (longitudinal) direction. Notwithstanding that these accelerations also occur in the mediolateral and anteroposterior directions, and given the bouncing mechanism of running, a larger muscular intervention would be needed to maintain motion of the CoM. This larger intervention relative to core muscular strength may be a cause or an effect of an underlying mechanically inefficient running gait that is then rendered potentially more noticeable by the inclusion of prior cycling. Instructing a triathlete to reduce longitudinal acceleration may be a viable training intervention. Indeed, cueing to reduce vertical oscillation has been observed to reduce peak vertical ground reaction force (Adams et al., 2018). Whilst ground reaction force was not measured in our study, our findings that an increased magnitude of longitudinal acceleration occurred in the initial 1 km after Run_Aero_ may help inform coaches who wish to use wearable devices for running gait modification post cycling.

Although we focused on examining the trajectory of CoM accelerations in cycling and running using accelerometry outside of the laboratory environment, other studies (Cejuela et al., 2007; Hausswirth et al., 2010; Weich et al., 2020) have examined gait mechanics relative to additional kinematic and kinetic parameters. For instance, an increase in running cadence has been reported to be accompanied by reductions in peak hip adduction angle, hip external adduction and internal rotation moments that influence the magnitude of CoM accelerations (Heiderscheit et al., 2012). An influence of this kind could suggest a deviation from an individual triathlete's accustomed running cadence.

Aside from running cadence, another spatiotemporal strategy associated with an increase in running cadence is a shortened step length (Chapman et al., 2008; Schubert et al., 2014). A reduction in step length places the initial loading closer to the triathlete's CoM (Heiderscheit et al., 2012). This may be reflected by a lower magnitude of anteroposterior acceleration during running after cycling. However, our study was designed to measure the immediate effect of the magnitude and trajectory of CoM acceleration in cycling in a drops and aerodynamic position and the impact on running. Adopting any new running mechanics based on reducing CoM magnitudes may require short-term increases in metabolic demand. In this regard, running economy is a complex, multifactorial concept that represents the sum of various metabolic, cardiorespiratory, biomechanical and neuromuscular characteristics during submaximal running (Barnes and Kilding, 2015). Although the demands of field based cycling and running motion on the magnitude of CoM acceleration are not well-described, elite distance runners have been observed to have slightly less vertical oscillation (Cavagna et al., 2005). Notably, empirical evidence suggests that reducing vertical oscillation has a positive effect on running economy (Mountjoy et al., 2014). Thus, increased vertical oscillation and acceleration magnitude are likely to be negatively connected to running economy.

It is clear, however, that any changes in neuromuscular activity and control, and/or joint kinematics have an impact on CoM motion. Our findings suggest that changes to the bicycle-athlete system influence the magnitude of acceleration to varying capacities. Some clues exist as to whether the degree of influence of hand position, specific cadences or running distance may have a greater influence on CoM acceleration. These clues should be followed up by longitudinal prospective studies. Better coaching strategies may then be established for assessing CoM acceleration during the bicycle to run transition in Sprint Distance triathlon.

### Limitations

The current study is not without limitations including small sample size; however, the effect size analysis addressed this typical shortfall. As the level of triathlete used in the current study was recreational, we are limited in our ability to suggest the same findings would apply to beginner or professional level athletes as well.

## Conclusion

The magnitude of CoM acceleration analysis was used in the context of cycling as well as in combination with running after a change in hand position during cycling in Sprint Distance triathlon. The results obtained with this approach suggest that triathletes significantly varied their CoM acceleration based on cadence and hand position. Concerning Cycle_Aero_, total CoM acceleration was significantly lower compared to Cycle_Drops_ despite triathletes reporting higher ratings of perceived exertion. Concerning Run_Aero_, it was found that triathletes reduced their longitudinal CoM acceleration compared to Run_Drops_ and completed the 5 km in a quicker time. Overall RPE was significantly lower in Run_Aero_. Future studies should look to assess full kinematic, kinetic, and loading rate parameters associated with CoM accelerations and changes in spatiotemporal measures over a longer duration of cycling and running. Coaches may look to use wearable devices for CoM motion during field-based settings to monitor performance in both cycling and running after cycling.

## Data Availability Statement

The raw data supporting the conclusions of this article will be made available by the authors, without undue reservation.

## Ethics Statement

The studies involving human participants were reviewed and approved by Human Research Ethics Committee board (HREC 030317) of Charles Darwin University. The patients/participants provided their written informed consent to participate in this study.

## Author Contributions

SE: conceptualization and methodology, data curation, writing—original draft preparation, and project administration. DJ: software. SE, DR, JL, and DJ: formal analysis. DJ and DR: writing—review and editing. JL: supervision. All authors have read and agreed to the published version of the manuscript. All authors contributed to the article and approved the submitted version.

## Conflict of Interest

The authors declare that the research was conducted in the absence of any commercial or financial relationships that could be construed as a potential conflict of interest.

## Publisher's Note

All claims expressed in this article are solely those of the authors and do not necessarily represent those of their affiliated organizations, or those of the publisher, the editors and the reviewers. Any product that may be evaluated in this article, or claim that may be made by its manufacturer, is not guaranteed or endorsed by the publisher.
